# Core competency in palliative care among intensive care unit nurses: A latent profile analysis

**DOI:** 10.1111/nicc.70021

**Published:** 2025-03-20

**Authors:** Qin Guan, Xiaoling Zhu, Zhipeng Xue, Mengyun Peng

**Affiliations:** ^1^ Faculty of Nursing, Dali University Dali China; ^2^ Department of Nursing First Affiliated Hospital of Dali University Dali China; ^3^ School of Nursing, Suzhou Medical College of Soochow University Soochow University Suzhou China

**Keywords:** core competency, intensive care units, latent profile analysis, nurses, palliative care

## Abstract

**Background:**

Intensive care unit (ICU) nurses play a leading role in integrating palliative care into ICU practices, which requires them to possess professional and comprehensive palliative care core competencies.

**Aim:**

To explore the current status of ICU nurses' palliative care core competency and to examine the factors influencing different subgroups of core competency.

**Study Design:**

A quantitative, cross‐sectional study. A random sampling of 342 ICU nurses from five hospitals participated in this study from March to April 2024. A latent profile analysis (LPA) was conducted to identify subgroups based on the Palliative Care Nurses' Core Competences (PCNCC) scale. Differences between the variables, including sociodemographic characteristics, autonomous learning capacity, job satisfaction and subgroups, were explored using multivariate logistic regression. This cross‐sectional study used the STROBE checklist.

**Results:**

The mean score for palliative care core competency among ICU nurses was (58.96 ± 21.56). There were three different subgroups of palliative care core competency, namely, the ‘low palliative care core competency group (31.2%)’, the ‘medium palliative care core competency group (47.2%)’ and the ‘low palliative care core competency group (21.6%)’. Professional title (odds ratio [OR] = 0.161, 95% confidence interval [CI]: 0.038–0.673, *p* = .012), position (OR = 0.111, 95% CI: 0.013–0.975, *p* = .047), work experiences (OR = 0.169, 95% CI: 0.030–0.965, *p* = .046) and autonomous learning capacity (OR = 3.298, 95% CI: 1.390–7.822, *p* = .007) were significant factors affecting the medium‐level group, while position (OR = 0.101, 95% CI: 0.011–0.918, *p* = .042) and autonomous learning capacity (OR = 3.878, 95% CI: 1.447–10.396, *p* = .007) significantly influenced the low‐level group.

**Conclusions:**

The majority of ICU nurses were categorized in the low and medium‐level palliative care core competency group; professional title, position, work experience and autonomous learning capacity were the main influencing factors.

**Relevance to Clinical Practice:**

ICU nurses should receive specific knowledge and training on palliative care, especially young nurses with limited work experience. Nursing managers and educators should provide targeted intervention strategies for nurses with different autonomous learning capacities to improve their core competencies in palliative care.

**What is known about the topic**

- Palliative care is widely regarded as an essential component of providing complete critical care to critically ill patients and their families.
- Nurses play a key role in facilitating the integration of palliative care into intensive care unit (ICU) practice and require comprehensive and specialized core competencies.
- Palliative care training and education for ICU nurses is lacking and core competencies are unclear.

**What this paper adds**

- The majority of ICU nurses were categorized into low‐and medium‐palliative care core competency groups.
- Different factors were found to affect different palliative care core competency subgroups among ICU nurses. Therefore, targeted intervention strategies for nurses should be implemented to improve core competency and meet the palliative care needs of patients and families.
- This study may provide insight into the global health care system's drive to integrate palliative care into the ICU and other departments.

## 1 INTRODUCTION

The goal of intensive care is to maintain essential functions to prevent death in patients with critical illnesses. Despite progress in technology and treatment, the risk of mortality because of infections in intensive care units (ICUs) remains high.^1^ Palliative care is commonly used in ICU decision‐making when life‐sustaining treatments do not suit the needs of patients at a high risk of mortality or when the burden outweighs the benefit.^2^ The International Association for Hospice and Palliative Care (IAHPC) developed a consensus‐based definition as follows: palliative care is active and holistic care for all people who are experiencing significant health‐related suffering because of critical illnesses, especially in terminally ill patients. It aims to improve the quality of life of patients, their families and caregivers.^3^ Palliative care is widely regarded as an essential component in providing complete critical care to critically ill patients and their families.^4^ Previous studies have indicated that nurses must take the lead in successfully integrating palliative care into critical care practices.^4^, ^5^, ^6^ This requires ICU nurses to have comprehensive and specialized core competency in palliative care.

## 2 BACKGROUND

Based on Prahalad and Hamel^7^ competency and core competency are different concepts. A core competency must make a significant contribution to patients benefits, and it cannot be imitable because it is deeply rooted in an individual's unique knowledge, skills and experience. Yang^8^ defined core competency as the effective integration of organizational knowledge, resources and skills. In ICU nursing practices, palliative care core competency for nurses includes pain management, symptom management, end‐of‐life discussions with patients/families, bereavement support and palliative care consulting.^4^, ^9^ Nursing core competency is an indicator of capability to completed tasks from professional perspective, which may affect interprofessional work, patient safety and quality of nursing.^9^ However, previous studies showed that the ICU nurses' preparedness for implementing palliative care is insufficient because of a lack of knowledge and competency, which is identified as an important barrier to palliative care.^10^, ^11^ For the goal of improved end‐of‐life care and palliative care to be realized, nurses must have the knowledge and tools to effectively provide care to individuals with life‐threatening illnesses. The International End‐of‐Life Nursing Education Consortium Training Programme (ELNEC) is a national education initiative to improve end‐of‐life care in the United States, and its viability is gradually being demonstrated globally.^12^ However, because of the influence of traditional Chinese culture, most critically ill patients and their families prefer to receive aggressive treatment even if the survival rate is low, resulting in the fact that palliative care is still in the early stages in mainland China and palliative care education is insufficient.^13^ Therefore, in order to facilitate the integration of palliative care into ICU, there is a need to explore the palliative care core competency level among ICU nurses.

It is well known that the core competency of nurses is influenced by a variety of factors. Chen et al.^14^ classified the main factors affecting gerontological nurse specialists' core competencies into three categories: individual‐level factors, employer‐level factors and training‐related factors. Li et al.,^15^ found that sociodemographic information, such as age, title, marital status and working years, significantly affects emergency‐room nurses core competencies. Wang et al.^16^ identified relationship quality, specialist training and nursing research abilities as key factors influencing core competency in midwifery. In additional, job satisfaction, a common independent variable, was often shown to be related to nurses' core competencies, as it may influence their commitment to learning.^14^ Ortega‐Lapiedra et al.^17^ conducted a concept analysis and showed that autonomous learners tend to exhibit higher competence in nursing. Although the evidence above suggested that nurses core competencies are influenced by diverse factors, the potential factors affecting ICU nurses' core competency in palliative care remain unclear.

Benner's theory of nursing competency development suggests that nurses' stages of competency evolve over time and experience, and that the acquisition of nursing competency is a continuous process. Furthermore, subgroups of nursing competency at different levels may be associated with different factors.^18^ A simplified grading that relies solely on total scores to stratify palliative care core competency may overlook individual‐level differences across subgroups. Latent profile analysis (LPA) is an individual‐centred approach that uses continuous explicit variables to cluster data, allowing for the exploration of group heterogeneity.^19^, ^20^ Unlike conventional variable‐centred methods, which disregard individual experiences, the individual‐centred approach focuses on identifying potential population subgroups based on multiple observed characteristics, offering higher specificity.^19^

## 3 AIMS AND OBJECTIVES OF STUDY

Therefore, the present study aims to explore the current status of ICU nurses' palliative care core competency, identify subgroups of core competency and examine influencing factors for different subgroups using LPA. This study posed the following research questions:

1. How many categories of palliative care core competencies among ICU nurses can be classified?
2. What are the characteristics of the ICU nurses with low and medium palliative care core competency groups?
3. How do demographic characteristics, autonomous learning capacity and job satisfaction influence different subgroups in ICU nurses?

## 4 DESIGN AND METHODS

### 4.1 Setting and samples

A quantitative, cross‐sectional study design was used. In five hospitals across four provinces in China, a convenient sample of ICU nurses was gathered between March and April of 2024. If a registered nurse had at least one year of hospital work experience and gave their agreement to participate, they were considered included. Larger sample sizes in surveys are known to yield more accurate and representative results. According to MacCallum et al.,^21^ there should be a minimum required sample size of 100 or a minimum sample size to variable number ratio of 5. Finally, four hundred nurses participated, and 342 completed the survey, providing a response rate of 85.5%.

### 4.2 Data collection tools and methods

#### 4.2.1 Demographic information

To better understand the factors influencing ICU nurses with low levels of palliative core competency, a demographic information was designed that included age, gender, marital status, education status, position, region, average family income per month, professional title, position, work experience and palliative care‐related education or training. This study also examined the impact of different levels of autonomous learning capacity (low, moderate and high) and job satisfaction (totally dissatisfied, dissatisfied, neutral, satisfied and completely satisfied) on core competency in palliative care, drawing on a previous study.^22^, ^23^

#### 4.2.2 Palliative Care Nurses' Core Competences scale

Palliative care nurses' core competence scale was developed by Han,^24^ which includes 28 items across four dimensions: ethical care competency (7 items), physical and mental care competency (10 items), spiritual care competency (6 items) and self‐psychological adjustment competency (5 items). Each item was assessed on a 5‐point Likert scale ranging from ‘not capable’ to ‘very capable’ for a total score of 0 to 112, higher scores indicating a higher core competency in palliative care. Palliative Care Nurses' Core Competences (PCNCC) in this study has a Cronbach's alpha of .924.

### 4.3 Data collection

This study was conducted on online, which is called ‘wenjuanxing’, the largest e‐questionnaire platform in China. One of the team members contacted the directors of the nursing departments of five hospitals in four provinces, and after explaining the study's purpose, process and obtaining consent, the directors of the nursing departments sent the questionnaire URL link to the participants who met the inclusion criteria. The first page of the questionnaire described the study in detail and asked participants if they were willing to join, and only those who selected ‘Yes, I agree to join’ were allowed to complete the rest of the questionnaire.

### 4.4 Data analysis

Profiles were found using LPA in Mplus 8.3. LPA is widely applied to estimate the number of subpopulations in an example. LPA can employ hypothetical categorical variables to clarify the relationship between exogenous continuous‐type indicators, allowing for the evaluation of the relationship between exogenous indicators while maintaining local independence among them.^25^ The log likelihood, Akaike information criterion (AIC), Bayesian information criterion (BIC), adjusted BIC, entropy, Lo‐Mendell‐Rubin (LMR) and bootstrapped likelihood ratio tests were employed to evaluate model fit and identify the optimal number of categories. To determine the best model, the models from each category's fitting results were mixed with the indicators.

The data were analysed using SPSS 26.0. For categorical data, this study utilized frequency and composition ratios; for continuous data, mean and standard deviation was used if they passed the Kolmogorov–Smirnov Test, non‐normally distributed data were expressed using median (interquartile range) M (P25‐P75). In order to compare categorical variables between groups, the chi‐square test was performed. The analysis of variance (ANOVA) was performed for assessing continuous variables across groups. A multivariate logistic regression model was used to evaluate the differences in demographic characteristics. *p* < .05 indicates a statistically significant difference.

## 5 ETHICAL CONSIDERATIONS

The study was approved by The First Affiliated Hospital of Dali University (with the codes DFY20240403001). The purpose and process of the study were explained in the consent form, as well as the voluntary nature and anonymity of participation and the possibility of withdrawal.

## 6 RESULTS

### 6.1 Participants

The study included 342 ICU nurses, 301 of whom were female (88.0%). The nurses' mean age was 10.23 years (SD = 6.37). More than half of the nurse (80.1%) were undergraduates, with 74.9% married. Table 1 shows the detailed characteristics. There were statistically significant differences in palliative care core competency by ICU nurses' position, work experience, palliative care‐related education or training, autonomous learning capacity and job satisfaction (*p* < .05).

**TABLE 1:** Social‐demographic differences in palliative care core competency among intensive care unit nurses (*n* = 342).

Variables	*n* (%)	Core competency in palliative care (M ± SD)	*t*/*F*	*p*
Gender			0.893	.372
Male	41 (12.0)	61.78 ± 20.99		
Female	301 (88.0)	58.57 ± 21.64		
Age			0.734	.481
<30	106 (31.0)	59.94 ± 22.16		
30 ~ 40	204 (59.6)	59.11 ± 21.58		
>40	32 (9.4)	54.72 ± 19.54		
Marital status			0.879	.416
Single	80 (23.4)	61.11 ± 22.08		
Married	256 (74.9)	58.13 ± 21.37		
Divorced or widowed	6 (1.8)	65.67 ± 23.30		
Education status			2.649	.072
≤Junior college	29 (8.5)	67.72 ± 23.66		
Undergraduate	274 (80.1)	58.19 ± 21.31		
≥Postgraduate	39 (11.4)	57.85 ± 20.76		
Religion			0.313	.754
Yes	15 (4.4)	60.67 ± 24.10		
No	327 (95.6)	58.88 ± 21.47		
Average personal income per month (yuan)			0.142	.868
<3000	35 (10.2)	60.74 ± 23.52		
3000 ~ 6000	173 (50.6)	58.90 ± 22.01		
>6000	134 (39.2)	58.57 ± 20.56		
Professional title			2.089	.125
Nurse	62 (18.1)	63.53 ± 24.03		
Senior nurse	126 (36.8)	59.17 ± 21.98		
Supervisor nurse or above	154 (45.0)	56.94 ± 19.96		
Position			2.378	.018
Clinical nurse	305 (89.2)	59.92 ± 33.10		
Head nurse	37 (10.8)	51.05 ± 14.37		
Work experiences (years)			3.158	.044
1 ~ 5	94 (27.5)	61.65 ± 22.64		
6 ~ 15	196 (57.3)	59.39 ± 21.67		
>15	52 (15.2)	52.48 ± 17.92		
Have you been received palliative care‐related education or training?			2.470	.014
Yes	78 (22.8)	64.22 ± 23.33		
No	264 (77.2)	57.41 ± 20.80		
Level of autonomous learning capacity			9.283	.000
Low	41 (12.0)	54.85 ± 20.30		
Moderate	244 (71.3)	57.11 ± 20.57		
High	57 (16.7)	69.81 ± 23.52		
Job satisfaction			4.513	.001
Totally dissatisfied	14 (4.1)	66.71 ± 22.11		
Dissatisfied	22 (6.4)	53.64 ± 19.79		
Neutral	130 (38.0)	53.82 ± 20.04		
Satisfied	155 (45.3)	62.14 ± 22.01		
Completely satisfied	21 (6.1)	67.67 ± 21.70		

### 6.2 Scores of core competency in palliative care among ICU nurses

Participants had a total palliative care core competency score of (58.96 ± 21.56) with a mean score of 2.11 for each item. Among the four dimensions of core competency, the mean score of ‘ethical care competency’ was rated as the highest 2.47, whereas ‘spiritual care competency’ had the lowest (1.69). The mean score of the ‘Physical and mental care competency’ and ‘Self‐psychological adjustment competency’ were between the other two dimensions (2.13 and 2.05, respectively).

### 6.3 Profile model selection

**FIGURE 1:**
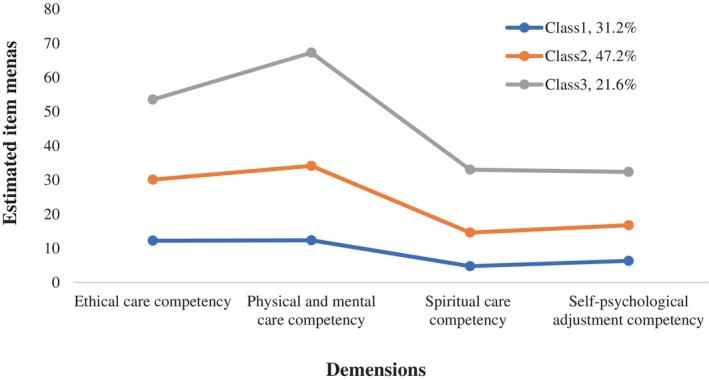
The latent profiles of the palliative care core competency dimensions.

Using the PCNCC Scale total score as the indicator, the model was fitted to possible profiles 1 through 5 in the present study. According to the *p*‐values for LMR and bootstrap likelihood ratio test (BLRT), profile model performs better as the profile increased. Conversely, because of the decreased entropy, the five‐profile model failed to perform better than the four‐profile model. Nevertheless, Table 2 indicates that the four‐profile model's probability of the class 2 was less than 5%. With an entropy of 0.894 and *p*‐values for both LMR and BLRT less than .05, the three‐profile model was found to be the best‐suited model with high classification certainty, taking into account the previously mentioned data (Figure 1).

**TABLE 2:** Model fitting indexes for LPA in core competency in palliative care (*n* = 342).

Model	AIC	BIC	ABIC	LMR (*p*)	BLRT (*p*)	Entropy	Probabilities of classes
1	8753.510	8784.189	8758.811				100%
2	8167.797	8217.650	8176.411	0.000	0.000	0.872	68.4%/31.6%
3	7902.506	7971.533	7914.433	0.000	0.000	0.894	31.2%/47.2%/21.6%
4	7791.786	7879.986	7807.025	0.003	0.000	0.905	45.3%/4.8%/29.8%20.1%
5	7731.719	7839.094	7750.271	0.024	0.000	0.889	12.9%/25.5%38.4%/4.6%/28.6%

Abbreviations: aBIC, sample‐size‐adjusted Bayesian information criterion; AIC, Akaike information criterion; BIC, Bayesian information criterion; BLRT, bootstrap likelihood ratio test; LMR, Lo‐Mendell‐Rubin adjusted likelihood ratio test.

Class 3 presented the highest palliative care core competency (named the ‘high palliative care core competency group’). Class 2 showed medium level of scores (named the ‘medium palliative care core competency group’). Class 1 had the lowest scores (named the ‘low palliative care core competency group’).

Multivariate logistic regression result was showed in Table 3. The profiles of palliative care core competency were used as the dependent variable, with the ‘high palliative care core competency group’ as the reference. Demographic information, autonomous learning capacity and job satisfaction served as independent factors in the analysis. The findings revealed a statistically significant effect of position (odds ratio [OR] = 0.101, 95% confidence interval [CI]: 0.011–0.918, *p* = .042) and autonomous learning capacity (OR = 3.878, 95% CI: 1.447–10.396, *p* = .007) on the low palliative care core competency group. Professional title (OR = 0.161, 95% CI: 0.038–0.673, *p* = .012), position (OR = 0.111, 95% CI: 0.013–0.975, *p* = .047), work experiences (OR = 0.169, 95% CI: 0.030–0.965, *p* = .046) and autonomous learning capacity (OR = 3.298, 95% CI: 1.390–7.822, *p* = .007) all had a statistically significant effect on the medium palliative care core competency group.

**TABLE 3:** Results of multivariate logistic regression analysis (*n* = 342).

Variable	Low palliative care core competency group (ref. high palliative care core competency)						Medium palliative care core competency group (ref. high palliative care core competency group)					
	*β*	SE	OR	95%CI		*p*	*β*	SE	OR	95%CI		*p*
				Lower	Upper					Lower	Upper	
Gender (ref. female)												
Male	−.352	0.515	0.703	0.256	1.931	.495	−.482	0.477	0618	0.243	1.573	.313
Age (ref. >40)												
<30	1.503	1.232	4.494	0.402	50.242	.222	2.143	1.152	8.523	0.892	81.474	.063
30 ~ 40	.643	1.071	1.903	0.233	15.526	.548	.957	0.991	2.604	0.373	18.166	.334
Marital status (ref. divorced or widowed)												
Single	.660	1.471	1.934	0.108	34.545	.654	.080	1.206	1.084	0.102	11.528	.947
Married	.931	1.387	2.538	0.167	38.504	.502	−.031	1.118	0.970	0.108	8.673	.978
Education status (ref. ≥postgraduate)												
≤Junior college	−1.475	0.855	0.229	0.043	1.223	.085	−.881	0.770	0.414	0.092	1.875	.253
Undergraduate	−.823	0.620	0.439	0.130	1.480	.184	−.912	0.584	0.402	0.128	1.263	.119
Religion (ref. no)												
Yes	.441	0.855	0.229	0.293	8.236	.605	−.227	0.841	0.797	0.153	4.141	.787
Average personal income per month (yuan) (ref. >6000)												
<3000	.027	0.665	1.028	0.279	3.780	.967	−.753	0.646	0.471	0.133	1.671	.244
3000 ~ 6000	.256	0.410	1.292	0.579	2.885	.532	.154	0.382	1.167	0.551	2.469	.686
Professional title (ref. supervisor nurse or above)												
Nurse	−.806	0.735	0.447	0.106	1.887	.273	−1.827	0.730	0.161	0.038	0.673	.012
Senior nurse	−.037	0.449	0.967	0.400	2.322	.934	−.244	0.419	0.783	0.344	1.782	.560
Position (ref. head nurse)												
Clinical nurse	−2.297	1.128	0.101	0.011	0.918	.042	−2.199	1.109	0.111	0.013	0.975	.047
Work experiences (years) (ref. >15)												
1 ~ 5	−1.718	1.126	0.179	0.020	1.631	.127	−1.342	1.061	0.261	0.033	2.090	.206
6 ~ 15	−1.687	0.950	0.185	0.029	1.191	.076	−1.776	0.888	0.169	0.030	0.965	.046
Have you been received palliative care‐related education or training? (ref. no)												
Yes	−.396	0.384	0.673	0.317	1.430	.303	−1.067	0.372	0.344	0.166	0.714	.004
Level of autonomous learning capacity (ref. high)												
Low	2.075	0.714	7.964	1.965	32.280	.004	1.585	0.660	4.881	1.340	17.777	.016
Moderate	1.355	0.503	3.878	1.447	10.396	.007	1.193	0.441	3.298	1.390	7.822	.007
Job satisfaction (ref. completely satisfied)												
Totally dissatisfied	−.160	1.106	0.852	0.097	7.452	.885	−.100	0.939	0.905	0.144	5.701	.915
Dissatisfied	.796	1.029	2.216	0.295	16.667	.439	.057	0.937	1.059	0.169	6.649	.951
Neutral	.863	0.796	2.370	0.498	11.270	.278	.061	0.693	1.063	0.273	4.131	.930
Satisfied	−.119	0.767	0.888	0.198	3.989	.877	−.400	0.653	0.671	0.186	2.412	.541

Abbreviations: CI, confidence interval; OR, odds ratio.

## 7 DISCUSSION

To our knowledge, this is the first study to explore clusters of ICU nurses with palliative care core competency. Using LPA, the study identified three groups according to ICU nurses' overall palliative care core competency ratings: the ‘high palliative care core competency group’ (31.2%), the ‘medium palliative care core competency group’ (47.2%) and the ‘low palliative care core competency group’ (21.6%). Among these, the ‘medium palliative care core competency group’ accounted for the majority. Although different measurement tools were used, our findings are consistent with the results of Kurnia et al.^11^ and Hamdan et al.^26^

The low and medium palliative care core competency groups accounted for nearly 80%, suggesting that the overall core competency in palliative care of ICU nurses in this study was at a lower‐middle level. This may be attributed to the serious condition of ICU patients and the uncertainty of disease progression, which increase nurses' workload. The limited time available for professional development makes it challenging for nurses to improve their palliative care core competencies. Additionally, the late development of palliative care practices in China, influenced by traditional cultural beliefs, has resulted in a lack of professional education and training related to palliative care.^27^ This may result in poor palliative care knowledge and behaviours.^28^ According to the classification of LPA, the scores of the four dimensions of the Class 3, namely, the ‘high palliative care core competency group’, are higher than those of the medium and low palliative care core competency groups, especially in the physical and mental care competency dimension. This indicates that physical and mental care ability, as a necessary routine practice skills for nursing staff, is more significant in the high palliative care core competency group. However, the scores of the four dimensions of the low palliative care core competency group are at the lowest level, which should attract the attention of nursing managers because it may affect the quality of care and patient safety. Previous study^9^ advocated that all the ICU team members should keep staff palliative care competencies to promote the integration of palliative care into ICU. According to the three‐class model in our results, nursing managers should provide different core competency development models for ICU nurses at different class, conducting regular training sessions on palliative care principles, communication skills and ethical decision‐making to help nurses master the specialized competency of palliative care.

This study showed that professional title and work experience are significant factors affecting the medium palliative care core competency group. Compared with high professional titles such as supervisor nurse or above, staff nurses in the medium palliative care core competency group have lower core competency (OR = 0.673, *p* = .012). This is consistent with Alan et al.^29^ who showed that the higher the professional title, the stronger the core competency of nurses. ICU nurses with higher professional titles and longer work experience often mean a high level of nursing skills and greater opportunities to receive palliative care education and training, which may enhance their core competencies. Previous studies^30^, ^31^ showed that nurses with high professional titles have a stronger desire for continuing professional development, which may promote their active learning of professional knowledge to improve their competency. Nursing managers should help ICU nurses with high professional titles to develop plans to achieve career goals and encourage nurses to pursue certifications in palliative or hospice care. For ICU nurses with junior professional titles and limited work experience, managers should fully mobilize their learning enthusiasm and increase the emphasis on implementing palliative care practices in the ICU. In addition, a spiritual care training protocol^32^ and fostering emotional resilience can be effective intervention strategies to help nurses improve competency in palliative care.

Position and autonomous learning capacity were important factors that affected the low and medium palliative care core competency groups found in this study. Compared with head nurses, clinical nurses in the low (OR = 0.918, *p* = .042) and medium (OR = 0.975, *p* = .047) palliative care core competency groups had lower core competency, which is consistent with Nabizadeh‐Gharghozar et al.'s (2021) study. In Benner's theory of competency development, nurses' competency development is divided into five stages, and only through clinical practice can they continuously enrich nurses' experience and knowledge.^18^ Nurse leaders are selected based on their clinical expertise; thus, ICU head nurses may perform better in palliative care core competency. In this study, compared with ICU nurses with high autonomous learning capacity, nurses with lower autonomous learning capacity in the low (OR = 10.396, *p* = .007) and medium (OR = 7.822, *p* = .007) palliative care core competency groups had significantly lower core competencies. Autonomous learning capacity as a key indicator for evaluating individual professional ability,^33^ should be viewed as a dynamic, ongoing and lifelong process by nurses as important from members of the health care team. ICU nurses with high autonomous learning awareness and capacity often have better decision‐making abilities and higher levels of engagement in clinical work and learning, which contributes to learning outcomes and competency development.^34^ On the one hand, ICU nurses should cultivate independent and autonomous learning capacity and value the essential of this capacity in the development of professional competencies. On the other hand, nursing managers should combine nurses' autonomous learning capacity to allocate a fixed amount of palliative care training, stimulate nurses with low autonomous learning capacity to learn and improve nurses' core competency in palliative care.

## 8 STRENGTHS AND LIMITATIONS

This study was designed with careful consideration to ensure quality and rigour. The sampling method was randomized to minimize selection bias, and the sample size was determined based on a calculation formula to enhance reliability. All the instruments were pre‐validated to ensure accuracy. Moreover, the study design adhered to established guidelines (e.g., STROBE statement), which are widely accepted in this research field. Despite these efforts, the study acknowledges potential limitations. Firstly, all participants were invited by the nursing leaders, and organizational limitations should be taken into account. Secondly, the data were collected via self‐reported online surveys, and recall bias may exist. Thirdly, this study's findings are exploratory and only provide an overview of the situation for ICU nurses, the validity must be evaluated further. Finally, even though the participants in this study were from different provinces, they were not representative of all ICU nurses, and the results are limited in generalization. ICU nurses' core competencies in palliative care may fail to change with self‐directed learning and organizational training; therefore, a longitudinal study was suggested to examine changes in different core competency groups.

## 9 IMPLICATIONS AND RECOMMENDATIONS FOR PRACTICE

As we know, professional title, position and working experiences reflect nurses' level of knowledge, skills and experience; therefore, nursing managers should differentiate between the varying needs for training in palliative care and the clinical application of professionally experienced and inexperienced ICU nurses. Improving the palliative care core competency among ICU nurses with low autonomous learning capacity requires targeted strategies that provide structured, engaging and supportive learning environments, such as mentorship and peer learning, while ICU nurses with high autonomous learning capacity are self‐motivated and proactive in seeking knowledge. To enhance their palliative care core competency, strategies should leverage their independence, critical thinking skills and adaptability.

## 10 CONCLUSION

Palliative care core competency plays a significant role in integrating palliative care into ICU practice. The majority of ICU nurses were categorized in the low and medium‐level palliative care core competency group; professional title, position, work experiences and autonomous learning capacity were the main influencing factors. These results provide new insights for nursing managers and educators to provide targeted intervention strategies for ICU nurses with different autonomous learning capacities to improve their palliative care core competency.

## PATIENT CONSENT STATEMENT

Consent was embedded at the beginning of the online questionnaire, and participants were prompted to withdraw at any time. Participants completed and submitted the electronic questionnaire, indicating that their informed consent had been obtained.

## FUNDING INFORMATION

This study was supported by the Research Foundation for the Doctoral Program of Dali University (DFY20220302) and the Yunnan Provincial Department of Education project (2024J0847).

## ETHICS STATEMENT

Ethics approval was obtained from The First Affiliated Hospital of Dali University (with the codes DFY20240403001) on 1 March 2024.

## Data Availability

The data that support the findings of this study are available on request from the corresponding author. The data are not publicly available due to privacy or ethical restrictions.
